# Contrasting glacier responses to recent climate change in high-mountain Asia

**DOI:** 10.1038/s41598-017-14256-5

**Published:** 2017-10-20

**Authors:** Akiko Sakai, Koji Fujita

**Affiliations:** Graduate School of Environmental Studies, Nagoya University, Nagoya, Japan

## Abstract

Recent studies of Asian glaciers have shown that glaciers in eastern Karakoram and West Kunlun have been slightly gaining mass while those in nearby Jammu Kashmir and Himalayas are losing mass, at rates of more than 0.5 m w.e.yr^−1^ and about 0.3 m w.e.yr^−1^, respectively. Two possible explanations have been proposed for this difference in glacier behaviour: spatial heterogeneity in climate change (climatic forcing) or differing glacier responses to climate change (glacier response). However, neither explanation has strong supporting evidence. Here, we examine the glacial response by calculating the mass-balance sensitivity to temperature change in high-mountain Asia. In support of the glacier-response explanation, we find a strong correlation between observed glacier surface-elevation changes and mass-balance sensitivity of glaciers. The high coefficient of determination (R^2^ = 0.61) suggests that spatially heterogeneous mass-balance sensitivity has more explanatory power than regionally different climate change for the recent contrasting glacier fluctuations in the high mountain Asia.

## Introduction

**Figure 1:**
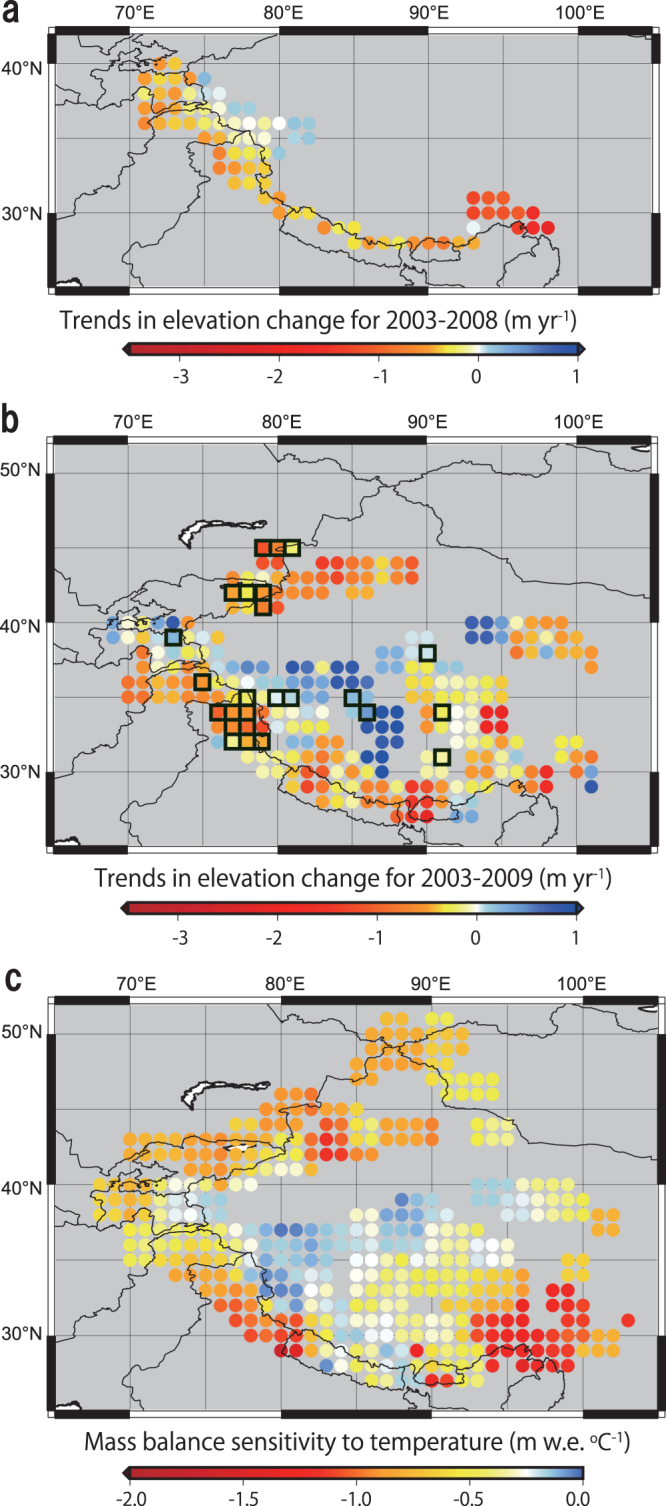
Trends in elevation change (TECs) and mass-balance sensitivity to air temperature change over high-mountain Asia (HMA). (**a**) TECs for glaciers along the Pamir-Karakoram-Himalaya for 2003–2008 modified from Kääb *et al*.^2^. Ref.^2^ is licensed under a Creative Commons Attribution 3.0 Unported License (https://creativecommons.org/licenses/by/3.0/). (**b**) TECs for glaciers in HMA for 2003–2009^1^ modified from Gardner *et al*., Science 340: 6134 (2013). Reprinted with permission from AAAS. Black square indicates data with high overlapping ratios (>0.8, see Methods). (**c**) Calculated mass-balance sensitivity (MBS) to temperature change. TEC values were calculated from ICESat and SRTM data. Original TEC data from ref.^1^ is the spatial average of a minimum of 50 TEC observations within a 50-km radius. All data are on a 1° grid, each point is the average over 2° cells (see Methods). These figures were created using The Generic Mapping Tools (http://gmt.soest.hawaii.edu/), Version 5.1.0.^30^ and were edited using Adobe Illustrator CS6 Version 16.0.0.

In recent years, shrinking glaciers have contributed to about 30% of global sea level rise^1^. Particularly in Asia, water demand exceeds supply due to rapid population growth, with glacier meltwater being a crucial water resource. Recent studies^1–5^ using altimetry and repeat DEMs demonstrated that glaciers in the Himalayas and Hengduan Shan are losing mass, whereas glaciers in the Karakoram and West Kunlun are slightly gaining mass (Fig. 1a and b). The growth of the latter glaciers, called the ‘Karakoram anomaly’^6^, has been attributed to cooling summer temperatures and increasing winter precipitation^7,8^. Similarly, in the nearby Tibetan Plateau, a high-resolution meteorological dataset provided additional evidence for mid-latitude Westerlies control on glacier mass balance^9^. However, the effect of such a ‘spatial climate heterogeneity’ explanation has not been comprehensively quantified over the entire high mountain Asia (HMA) region, leaving the possibility that much of the anomaly may be due to different glacier responses to similar climatic variations^10^.

In the Karakoram and Himalayas, debris cover on glaciers produces a spatial variation in glacier fluctuations^11^. Moreover, the sensitivity of a glacier to changing climate depends on the glacier’s present environment, specifically, the environmental control on the heat and mass balances of glaciers^12–17^. For instance, glaciers in an arid environment require relatively colder conditions and tend to be less sensitive to temperature change than those in a warm and humid climate^12–15^. In addition, the seasonality of precipitation, which varies widely over the HMA region, will affect the mass-balance sensitivity of glaciers^16–20^. These studies suggest that glaciers can undergo different mass changes even under uniform climate change.

Despite these localized studies^7,21,22^, no large-scale analysis has explained the heterogeneous changes in Asian glaciers observed with remotely sensed data^1–5^. To understand the mechanism for simultaneous mass losses in the Himalayas and Hengduan Shan with slight mass gains in the Karakoram and West Kunlun, we calculated mass-balance sensitivity to air temperature (MBS) using the Glacier Area Mapping for Discharge from the Asian Mountains (GAMDAM) glacier inventory^23^ to evaluate the glacier response. Further, we examined the spatial distribution of trends of climate change using reanalysis data sets (see Methods) to evaluate climatic forcing. The results were compared with two measures of trends in elevation change (TEC)^1,2^, which is expressed as *Δh/Δt* in Eq. (1) in Methods. The MBS calculation also incorporated an energy- and mass-balance model^17^ that used optimized precipitation for each region as input^19^ (see Methods).

## Results

**Figure 2:**
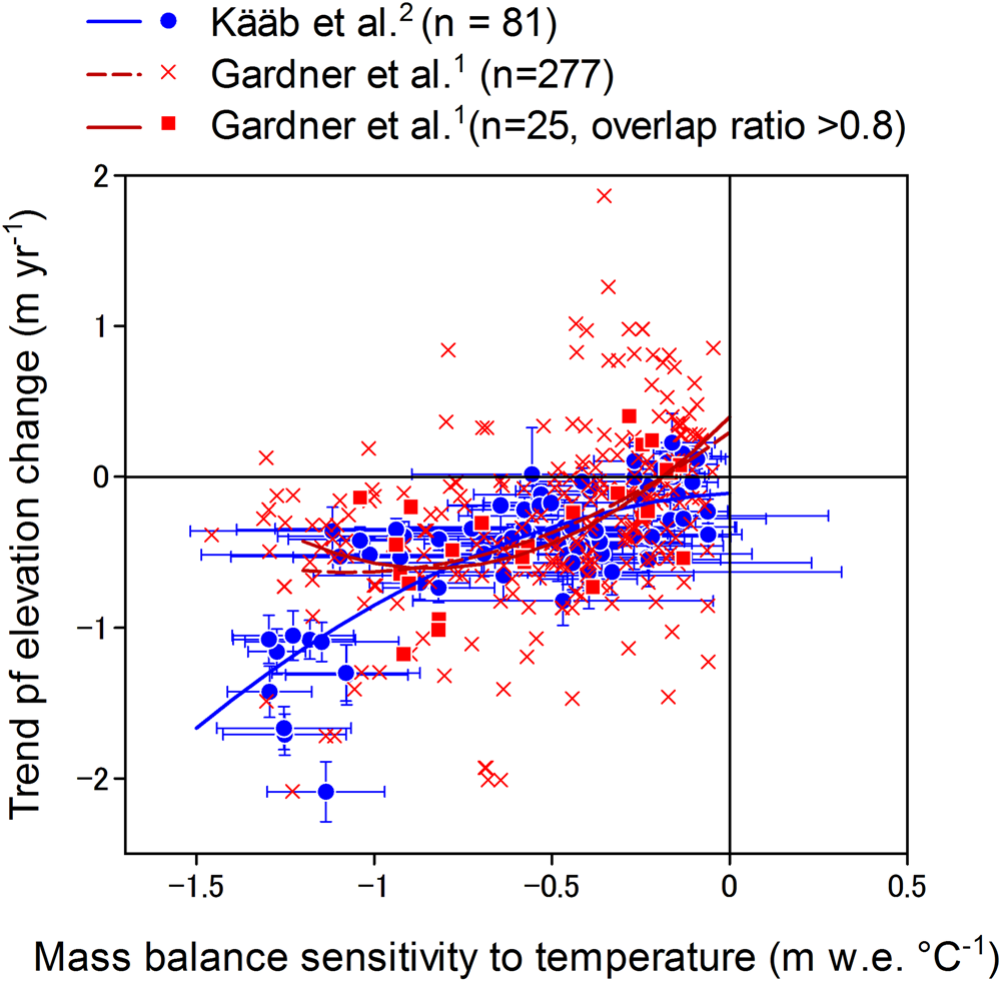
Relationship between the sensitivity of mass balance to temperature change and changes in TECs for glaciers^1,2^. Vertical error bars indicate the standard error calculated in ref.^2^, horizontal error bars indicate the standard deviation calculated from averaging each 0.5° grid point in the MBS calculation over a 2° area. There are three different TECs: Kääb *et al*.^2^, Gardner *et al*.^1^ with high overlapping ratios (>0.8), and Gardner *et al*.^1^ for all of HMA. Equations for regression lines and statistics are summarized in Table S1.

The resulting MBS distribution (Fig. 1c) is similar to the distribution of TECs obtained from remotely sensed laser altimetry observations of glacier surfaces (Fig. 1a and b). To quantify the correlation between MBS and TECs, we compare each value in Fig. 1a–c covering a 1° grid (each value is the average over the surrounding 2° × 2° region) in the scatter plot (Fig. 2, Table S1). From the first set of data^2^ (Fig. 1a), the coefficient of determination for the regression line is high (R^2^ = 0.61), suggesting that over 60% of the spatial variance in glacier mass balance is due to the spatially differentiated glacier response to temperature change. As a check, we also compared the relationship with the TEC dataset over a wider region from ref.^1^ (Fig. 1b) for two cases: limited to the same domain as in ref.^2^ and the entirety of HMA; we obtained lower, though consistent, correlations (R^2^ = 0.17 for the ref.^2^ domain and R^2^ = 0.19 for HMA). In particular, TECs from ref.^1^ at high MBS exhibit less change than those from ref.^2^, and have second order convex-downward regression curves (Fig. 2).

## Discussion

Optimized precipitation at the equilibrium line altitude (ELA), which is required to calculate MBS, was estimated using reanalysis datasets and an assumption that median glacier elevation is equal to the ELA, i.e. the accumulation area ratio (AAR) is 0.5. Actual glaciers have fluctuating masses, and therefore, the ELA does not always correspond to the median elevation. Shrinking glaciers tend to have less AAR, and the actual ELA should be higher than the median elevation. Therefore, the optimized precipitation would be overestimated over regions with shrinking glaciers. However, the discharge calculated using optimized precipitation performed well and had less bias compared to observed discharge in the HMA^24^, suggesting that optimized precipitation is close to actual precipitation in glacier areas. We calculate errors in the MBS, which can be caused by uncertainty of air temperature and shortwave radiation in the reanalysis datasets. The uncertainties of air temperature (0.9 °C) and shortwave radiation (102 W m^−2^), which were obtained as root mean square errors (RMSEs) against in-situ observational data^19^, result in RMSEs of the MBS ranging 200–260 m w.e. °C^−1^ (Fig. S1). The errors due to shortwave radiation increase with more negative MBS while those due to air temperature seem to have no trend. Uncertainty in the ELA assumption, which was evaluated to be 71 m^19^, is equivalent to 0.43 °C with a temperature lapse rate of 6.0 °C km^−1^; roughly half of the RMSE of air temperature.

The lower correlation between MBS and TEC from ref.^1^ for the entire HMA, and the largely different TECs at high MBS likely arise from different glacier extents in the inventories used. The TEC study of ref.^1^ aimed to estimate glacier mass change in the HMA in addition to worldwide, excluding the Greenland and Antarctic ice sheets. Hence, they analysed TECs using version 2.0 of the Randolph Glacier Inventory (RGI2.0)^25^, which has some seasonal snowcover mistakenly catalogued as glaciers^23^. In comparison, the TEC study of ref.^2^ used the more carefully selected ICESat footprints for glacier surfaces. Therefore, we calculated overlapping ratios between glacier areas in the RGI2.0 and GAMDAM glacier inventory to total RGI2.0 for each grid cell (see Methods and Fig. 1b and S2). The TEC data from ref.^1^ were screened based on high overlapping ratios; only TEC data having high probability of ICESat footprints on glaciers were selected. We obtained a relatively higher coefficient (R^2^ = 0.42) between MBS and TECs using a high overlapping ratio threshold, ratios >0.8 were selected (Fig. 2). Selected data from ref.^1^ are located in the ref.^2^ domain and in the Tien Shan and Tibetan Plateau. This suggests that the high correlation between glacier mass changes (TECs) and glacier mass balance sensitivity to temperature (MBS) might apply to the whole HMA in addition to the domain defined in ref.^2^.

Other differences between these datasets include different methods for assembling data; ref.^2^ aggregated each data in 1° grids cell while ref.^1^ aggregated at erratic points covering a 50 km radius. In addition, each point data in Fig. 2 from refs^1,2^ have slightly different TEC coverages in ref.^1^. Furthermore, TECs were derived only from autumn ICESat campaigns in ref.^2^ but from both autumn and winter campaigns in ref.^1^.

Apart from uncertainties in MBS and TECs, the coefficient of determination with TECs from ref.^2^ predicts that 61% of the spatial difference in glacier behaviour is due to different MBS, i.e. glacier responses. Furthermore, trends in summer temperature and annual precipitation, which were analysed in terms of climatic forcing, have lower determination coefficients with TECs throughout the calculated period (1979–2007, Fig. S3). The maximum determination coefficients are found for the trends from 1993 to 2007 for summer temperature (R^2^ = 0.32) and from 1997 to 2007 for annual precipitation (R^2^ = 0.20); the distributions of trends (Fig. S4) are significantly different from those of MBS (Fig. 1c). Furthermore, periods exhibiting high correlations are different between the datasets from refs^1,2^ for trends in both summer temperature and annual precipitation. This suggests that the spatial heterogeneity of climate change is not a major contributor to the spatial heterogeneity in glacier mass change. TECs were obtained from 2003–2008 data^2^, and the highest correlations between TECs and trends in summer temperature and annual precipitation are found between the 1990s and 2007 suggesting temporal gaps between climate forcing and TECs. We find no significant correlation between TECs and trends in temperature or precipitation during the TEC analysis period. There might have been step-like fluctuations in temperature and/or precipitation before 2003, and then glaciers have responded with some delay.

We classified the MBS-TEC scatter plot (Fig. 2) into 10 sub-regions following ref.^2^, as shown in Fig. S5. In Figs 2 and S5b, variability in TEC (vertical variability in the figure) with the same order of MBS could imply a local variability in climatic forcing. For instance, glaciers in East Nyainquentanglha Shan have dominantly large absolute MBS and TEC values compared to other regions. East Nyainquentanglha Shan receives high precipitation^26^, and thus, the error in MBS might be large, as described previously. The difference in TECs between Karakoram and West Nepal can be explained with the difference in MBS (Fig. S5b). However, MBS values for the Everest region in Nepal, Pamir, and Hindu Kush are similar to those in Karakoram but the absolute values of TECs in the former regions are clearly greater than those in the latter. This difference may be due to the Karakoram glaciers having a relatively less negative mass change under favourable climate forcing that maintains the glaciers, such as cooling summer temperatures and/or increasing winter precipitation^7,8^. Glaciers in the former regions show relatively rapid mass loss due to warming temperatures and/or decreasing summer precipitation^27^. The ‘Karakoram anomaly’ has drawn attention in the recent decade because glaciers in the Karakoram Range do not appear to follow general global warming trends. The MBS-TEC plot (Fig. S5b), however, shows that the Karakoram glaciers are not outliers. Therefore, we conclude that the Karakoram glaciers are not behaving anomalously; they have an insensitive MBS, and thus change their mass slightly due to local climatic forcing. Glaciers in the West Kunlun Shan-Tarim region also have less negative MBS values while their TECs are relatively less negative, even positive, compared to those in other regions with similar MBS values (e. g. the Everest region, East Pamir, and north-east margin of Spiti Lahaul). These results suggest that cooling temperatures and/or increasing precipitation might contribute to the slight mass gain in the West Kunlun Shan-Tarim region.

**Figure 3:**
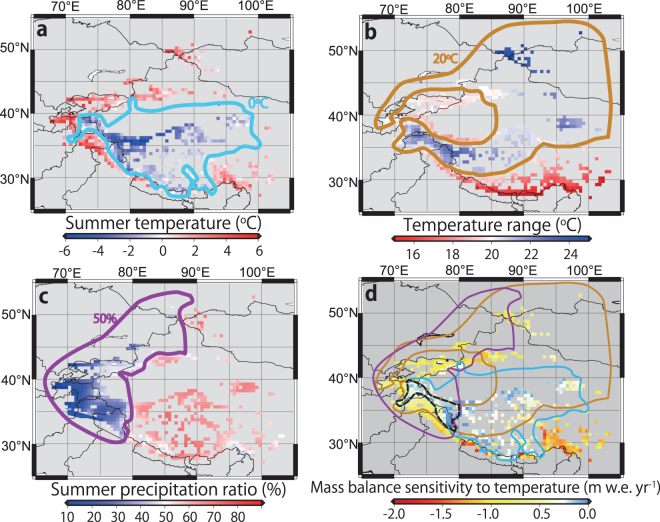
Distributions and boundaries of explanatory variables for multi-regression analysis and area boundaries for each explanatory variable with a weaker MBS. (**a**) The area with summer temperatures <0 °C is encompassed with a light blue line. (**b**) The areas with an annual range in monthly temperature >20 °C is encompassed with an orange line. (**c**) The area with a summer precipitation ratio of <50% is encompassed with a purple line. (**d**) MBS distribution showing overlapping areas of lower summer temperature (<0 °C), higher temperature ranges (>20 °C) and lower summer precipitation ratio (<50%) with a black dashed line. a-c are depicted based on a 0.5° grid cell. Each threshold for explanatory variables is provided in Fig. S6, which indicates that each threshold divides the MBS values such that half are less negative (weaker) and half are more negative (stronger). These figures were created using The Generic Mapping Tools (http://gmt.soest.hawaii.edu/), Version 5.1.0.^30^ and were edited using Adobe Illustrator CS6 Version 16.0.0.

Our multiple regression analysis reveals that the variance in MBS can be reduced by 69% with three explanatory variables: summer temperature, annual range of monthly temperature (temperature range), and ratio of summer precipitation (June-July-August) to annual (termed the summer precipitation ratio) (Table S2). Figure S6 and the determination coefficient with MBS (R^2^, Table S2) clearly show the impact of each explanatory variable on MBS values. Summer temperature shows a relatively simple and higher correlation with MBS (Fig. S6b) while the summer precipitation ratio has a largely varying relationship with MBS (Fig. S6c). In Fig. S6c, winter accumulation type glaciers (summer precipitation ratio <50%) have no strong sensitivity (MBS) while summer accumulation type glaciers (summer precipitation ratio >50%) have large variations in MBS. This difference is likely due to summer accumulation type glaciers having large variability in summer temperature (Fig. 3d), which has the strongest impact on MBS. Figure 3a–c show the area of each explanatory factor that corresponds to weaker (less negative) MBS; the relationships between MBS and explanatory factors are depicted in Fig. S6. Figure 3d shows that these overlapping areas with weak MBS tend to coincide with regions with small TEC, such as Karakoram and West Kunlun Shan (Fig. 1a and b). Conversely, regions with little or no overlap between the areas of weak MBS tend to show larger TEC, such as the Hengduan Shan, Bhutan, and western Nepal Himalayas (Fig. 3d). We conclude that climatic settings represented by the three factors, summer temperature, temperature range, and summer precipitation ratio, are the dominant control on heterogeneous mass balance sensitivity (MBS) and, consequently, the spatially contrasting mass change in Asian glaciers.

It should be noted that the MBS and multiple regression analyses performed in this study depend on quality of the reanalysis datasets. In this regard, the factors controlling mass-balance sensitivity to temperature (Table S2) also have crucial implications for projections of glacier mass change. Unrealistic seasonal patterns of air temperature and precipitation used for projected glacier mass changes would, for instance, lead to erroneous estimated changes even given accurate annual averages. In HMA, predicting the variability in monsoon extent will be a key factor in projecting glacier mass changes because changes in monsoon extent could alter the distributions of seasonal patterns of air temperature and precipitation.

## Methods

### Glacier response and climatic response in the calculation of glacier mass change

Water-equivalent mass change of glaciers can be expressed using elevation changes and glacier sensitivity as follows.

1 $$\frac{{\rm{\Delta }}M}{{\rm{\Delta }}t}=S\rho \frac{{\rm{\Delta }}h}{{\rm{\Delta }}t}=S(\frac{db}{dT}{\rm{\Delta }}T+\frac{db}{dP}{\rm{\Delta }}P)\,$$

where, *M*, *t*, *S*, *ρ*, *h*, *b*, *T*, and *P* are glacier mass, time, glacier area, density of snow or ice, glacier elevation, specific mass balance, temperature, and precipitation, respectively. *db*/*dt* and *db*/*dP* in eq. (1) indicate mass balance sensitivity to temperature (MBS) and precipitation, respectively, which are expressed as glacier responses to climate change. The spatial distribution of *ΔT* and *ΔP* are expressed as climate change.

In this study, we compare ${\rm{\Delta }}h/{\rm{\Delta }}t$ (TEC in units of m a^−1^) reported in refs^1,2^, and *db*/*dT* (MBS in units of m w.e. °C^−1^) in the eq. (1), as shown in Fig. 2.

Glacier ice is generally exposed at lower part of the glacier, while the upper glacier area is covered with accumulated snow. In the TEC analysis, we cannot obtain water-equivalent mass changes but trends in elevation changes. In eq. (1) there is some uncertainty in the density of snow or ice to estimate *ΔM*. ref.^2^ included the uncertainty in ice or snow density in the TEC error (±0.02 m a^−1^) by taking surface conditions (ice or snow) into account.

### Calculation of mass-balance sensitivity to temperature (MBS)

In the HMA, the Asian Precipitation Highly-Resolved Observational Data Integration Towards Evaluation (APHRODITE^28^) dataset was the most appropriate for precipitation^29^. However, the data has a particular bias at high altitude^19^ because the data was generated based on gauge data taken at primarily low altitude. We therefore optimized the precipitation amount after referencing the energy- and mass-balance condition on the glaciers. Previous studies^12,13^ established the relationship between summer mean air temperature and annual precipitation at the ELA based on observational data for glaciers worldwide; they suggested that there are quadric^12^ or power law^13^ relationships between these meteorological elements. For glaciers in HMA, although few glaciers are available, median elevations correspond well to observed ELA^19^. Furthermore, it has been confirmed that free atmosphere air temperature in the ERA-Interim global atmospheric reanalysis dataset^30^ correspond well to observed temperature at/around glaciers in HMA (root mean square error = 0.9 °C)^19^. We calculated glacier-area weighted average for median elevations in each 0.5° grid cell, and calculated the free atmosphere air temperature from the ERA-Interim^30^ at the median elevation. To obtain precipitation data on glaciers, we used an energy and mass balance model^16^.

Glacier mass balance (B) can be calculated as:

2 $$B={C}_{a}-{Q}_{M}/{l}_{m}+{E}_{V}+{R}_{F}$$

where *C*_*a*_, *Q*_*M*_/*l*_*m*_, *E*_*V*_, and *R*_*F*_ are accumulation, melt water, evaporation, and refreezing, respectively. *Q*_*M*_, and *l*_*m*_ are heat for ice melt and latent heat for melting ice. *C*_*a*_ is determined along with air temperature (snow or rain).

Heat for glacier melting (*Q*_*M*_) can be calculated using air temperature, relative humidity, wind speed, and solar radiation as:

3 $${Q}_{M}=(1-\alpha ){R}_{S}+{R}_{L}-\sigma {T}_{S}^{4}+{Q}_{S}+{E}_{V}{l}_{e}+{Q}_{G}$$

where *α*, *R*_*S*_, *R*_*L*_, *σ*, *T*_*S*_, *Q*_*S*_, *E*_*V*_ *l*_*e*_, *l*_*e*_, and *Q*_*G*_ are surface albedo, downward shortwave radiation, downward longwave radiation, the Stefan–Boltzmann constant, surface temperature in Kelvin, sensible heat flux, latent heat flux, latent heat for evaporation of water or ice, and conductive heat flux into glacier ice, respectively.

To calculate optimized precipitation at ELA (median elevation) (*P*_*opt*_), we assume that mass balance from 1979 to 2007 should be equal to 0 by adjusting the APHRODITE precipitation data as:

4 $${P}_{opt}={A}_{P}\,{P}_{AP}$$

where *A*_*P*_ is an adjusting ratio for APHRODITE precipitation, which is obtained differently in each grid cell. *P*_*AP*_, *P*_*opt*_, and summer mean temperatures at the averaged median elevation are plotted in Fig. S7. MBS is the mass balance change per one degree increase in air temperature at the ELA, which is calculated after changing only air temperature ± 0.5 °C from the equilibrium condition:

5 $${\rm{MBS}}=\frac{\bar{B}({T}_{a}+{\rm{\Delta }}T/2)-\bar{B}({T}_{a}-{\rm{\Delta }}T/2)}{{\rm{\Delta }}T}$$

where $\bar{B}$ is the average of annual mass balances for the period 1979–2007, and Δ*T* is 1 °C.

APHRODITE precipitation data (*P*_*AP*_)^28^ were plotted with annual precipitation in Fig. S7. The relationship shows that APHRODITE precipitation data has large variability, and there is no clear relationship between temperature and annual APHRODITE precipitation data at the ELA for Asian glaciers. We cannot obtain presumable MBS without bias correcting the APHRODITE precipitation data, because suitable precipitation and temperature data are required to estimate precise MBS^14^. The original APHRODITE precipitation (*P*_*AP*_) has less variability than the optimized precipitation (*P*_*opt*_), and no clear relation is found between summer mean air temperature and annual precipitation. On the other hand, the optimized precipitation shows large variability and its fitting curve becomes very close to a previously proposed approximation^12^. These results suggest that we can obtain plausible datasets for temperature and precipitation at the ELA, and therefore calculate reasonable MBS values.

Uncertainty of the ERA-Interim dataset, which could cause errors in MBS was obtained as root mean square errors of air temperature (0.9 °C) and shortwave radiation (102 W m^−2^) against *in-situ* observational data^19^. We first calculate optimized precipitation by changing air temperature or shortwave radiation by each RMSE, and then obtained each MBS (Fig. S1).

### Trends in elevation change

TECs (trends in elevation change) were analysed using ICESat data and the February 2000 SRTM (Shuttle Radar Topography Mission) DEM. The ICESat footprints were classified into glacier and non-glacier manually using Landsat images in Kääb *et al*.^2^, and RGI ver. 2.0^25^ in Gardner *et al*.^1^ TECs were then estimated at every 1° grid point (each value averaged over the surrounding 2° area) by fitting a robust linear trend to the time series of elevation differences between the SRTM DEM and individual ICESat footprint elevations.

To compare TECs and MBS, we calculated the area-weighted average MBS at each 1° grid covering the 2° grid-averaged glacier. TECs from Gardner *et al*.^1^, which were sorted at each arbitrary point covering 50 km in radius, were also aggregated at each 1° grid covering the 2° grid-averaged glacier.

### Overlapping area between the RGI2.0 and GAMDAM glacier inventories

The Randolph Glacier Inventory ver. 2.0 (RGI2.0) includes non-glacier area because source satellite images have seasonal snow cover. Recently, a glacier inventory covering the HMA, termed the GAMDAM glacier inventory (GGI), has been published^23^. Satellite images used to delineate glacier boundaries were carefully selected to avoid seasonal snow cover, and glacier areas were extracted manually after concurrently verifying with high resolution Google Earth images. Here we used the GAMDAM glacier inventory as a reference inventory, and calculated the overlapping ratio (*R*_*o*_) at each 0.5° grid cell to evaluate RGI2.0 as follows:

6 $${R}_{o}=\frac{{S}_{o}}{{S}_{rgi}}$$

where *S*_*o*_ and *S*_*rgi*_ are overlapping area between RGI2.0 and GGI and the glacier area of RGI2.0, respectively. The overlapping ratio indicates a probability that an ICESat footprint coincides with a glacier area in GGI (Fig. S2).

### Trends in climate forcing

To investigate trends in climate forcing, we conducted trend analyses of summer (June-July-August) temperature and annual precipitation using a reanalysis data set (ERA-Interim^30^) and gridded precipitation data (APHRODITE^28^). First, we calculated annual and summer data for each 0.5° grid, and then aggregated those data into each 1° covering 2° grid cell, weighted by glacier area. We further applied Sen’s trend to the annual data for each 1° grid cell from various starting years until 2007.

### Multiple regression analysis for mass-balance sensitivity to temperature

For the multiple regression analysis, we assumed summer temperature, annual precipitation, temperature range, summer precipitation ratio, and summer solar radiation at ELA as explanatory variables for MBS, as described in previous studies^10,12–19^, and prepared those data for each 0.5° grid cell. More details are provided in the Supplementary Materials (Table S2).

### Materials availability

All data needed to evaluate the conclusions in the paper are present in the paper and/or the Supplementary Materials. Additional data related to this paper may be requested from the authors.

## Electronic supplementary material

Supplementary Information
