# Supplementary material for: Contrasting glacier responses to recent climate change in high-mountain Asia
Source: Sci Rep. 2017 Oct 20;7:13717. doi: 10.1038/s41598-017-14256-5 (PMC5651914; doi:10.1038/s41598-017-14256-5)
Supplement: Supplementary file 1 — Supplementary Information [file 41598_2017_14256_MOESM1_ESM.pdf]

Supplementary information:

Contrasting glacier responses to recent climate change in high-mountain Asia

Akiko Sakai[1]* & Koji Fujita[1]

[1] Graduate School of Environmental Studies, Nagoya University, Nagoya, Japan.

*Correspondence author: E-mail: shakai@nagoya-u.jp

This file includes:

Figures S1-S7

Tables S1-S2

Supplementary Information

Figures:

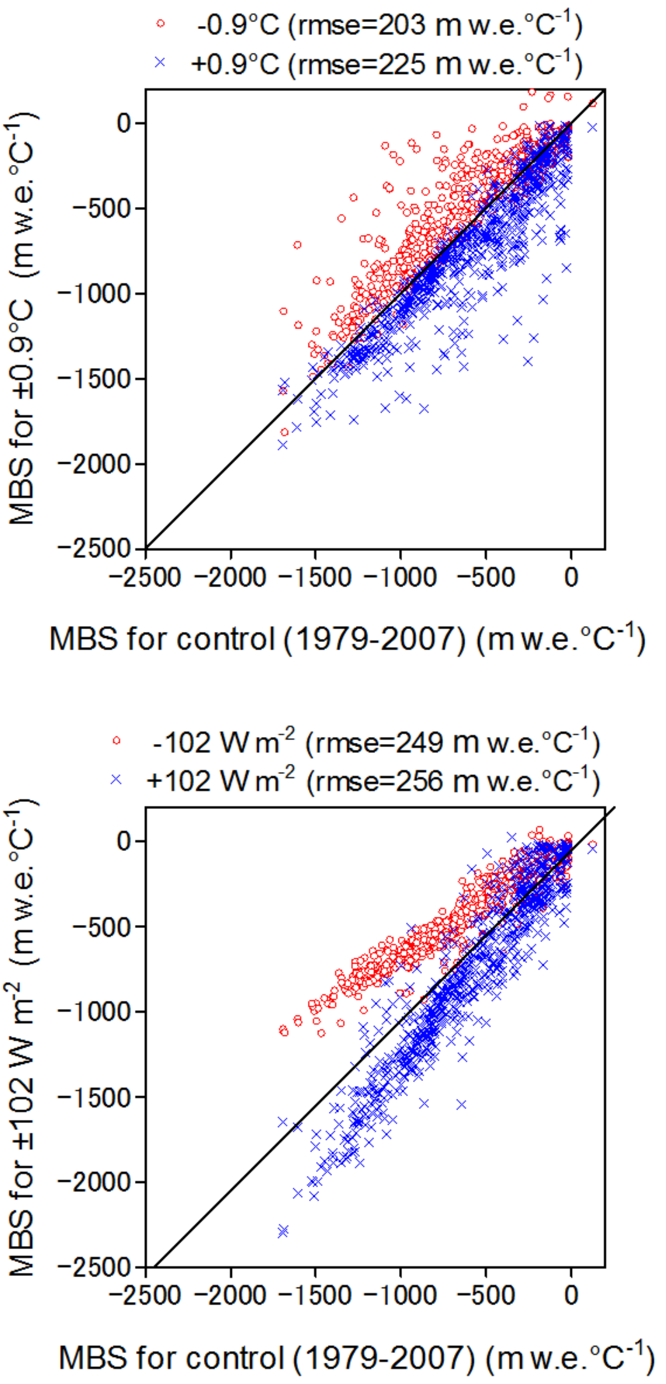

Fig. S1: Uncertainties of MBS. Relation between the control MBS and MBSs calculated by changing each RMSE of air temperature and shortwave radiation.

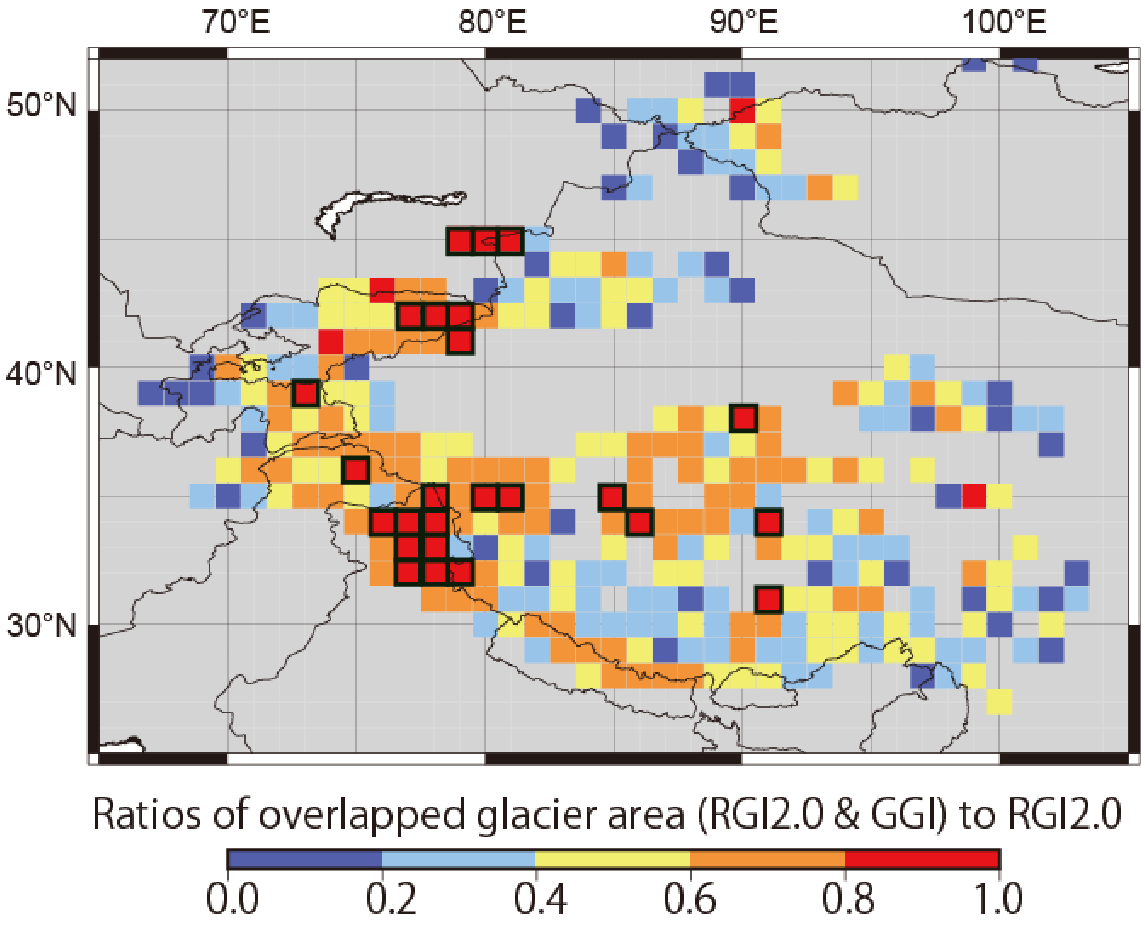

Fig. S2: Distribution of overlapping ratio, R_o. High overlapping ratios (> 0.8) framed with black lines indicate the grid cells for selected TEC data in Fig. 1b from Gardner. These figures were created using ‘Generic Mapping Tools' (http://gmt.soest.hawaii.edu/), Version 5.1.0. and were edited using Adobe Illustrator CS6 Version 16.0.0.

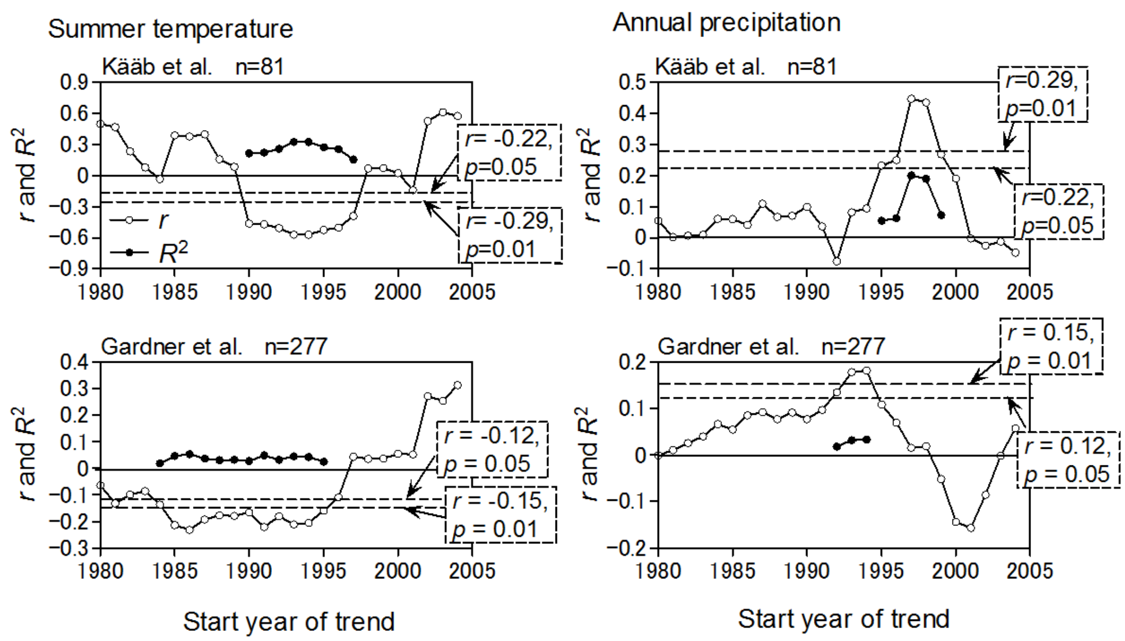

Fig. S3: Correlation coefficient (*r*) and coefficient of determination (R^2) of linear regression between TECs (Kääb et al.[2] and Gardner et al.[1]) and trends in summer air temperature and annual precipitation. The trends were calculated from each starting year to a fixed end year of 2007. Horizontal axes show starting years for trends until 2007. R^2 are indicated only when the significance levels (*p*) of correlation coefficients (*r*) are smaller than 5%, which are shown enclosed with dashed boxes.

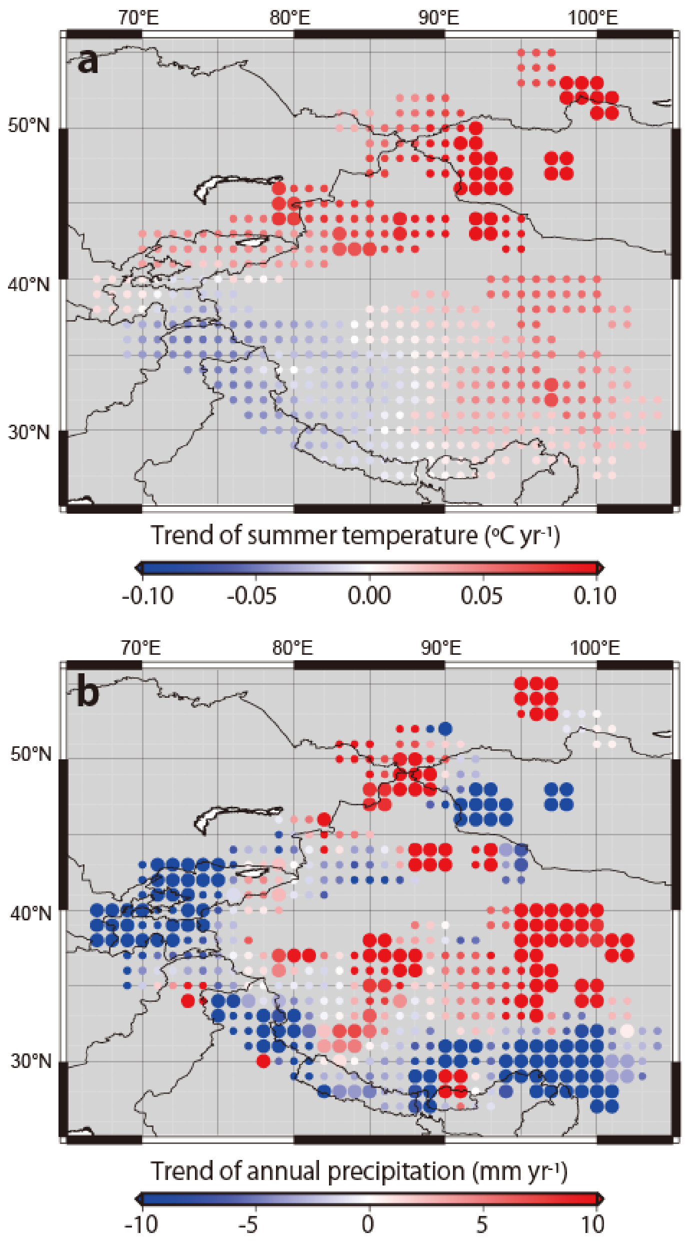

Fig. S4: Trends in **a.** summer temperature from 1993 to 2007 and **b.** annual precipitation from 1997 to 2007. These two periods are those with the highest coefficients of determination (R^2) with TEC from Kääb et al[2]. Large circles denote statistically significant trends estimated with the Mann-Kendall rank statistic and a significant level of 5%, while small circles have no statistically significant trends. These figures were created using 'The Generic Mapping Tools' (http://gmt.soest.hawaii.edu/), Version 5.1.0. and were edited using Adobe Illustrator CS6 Version 16.0.0.

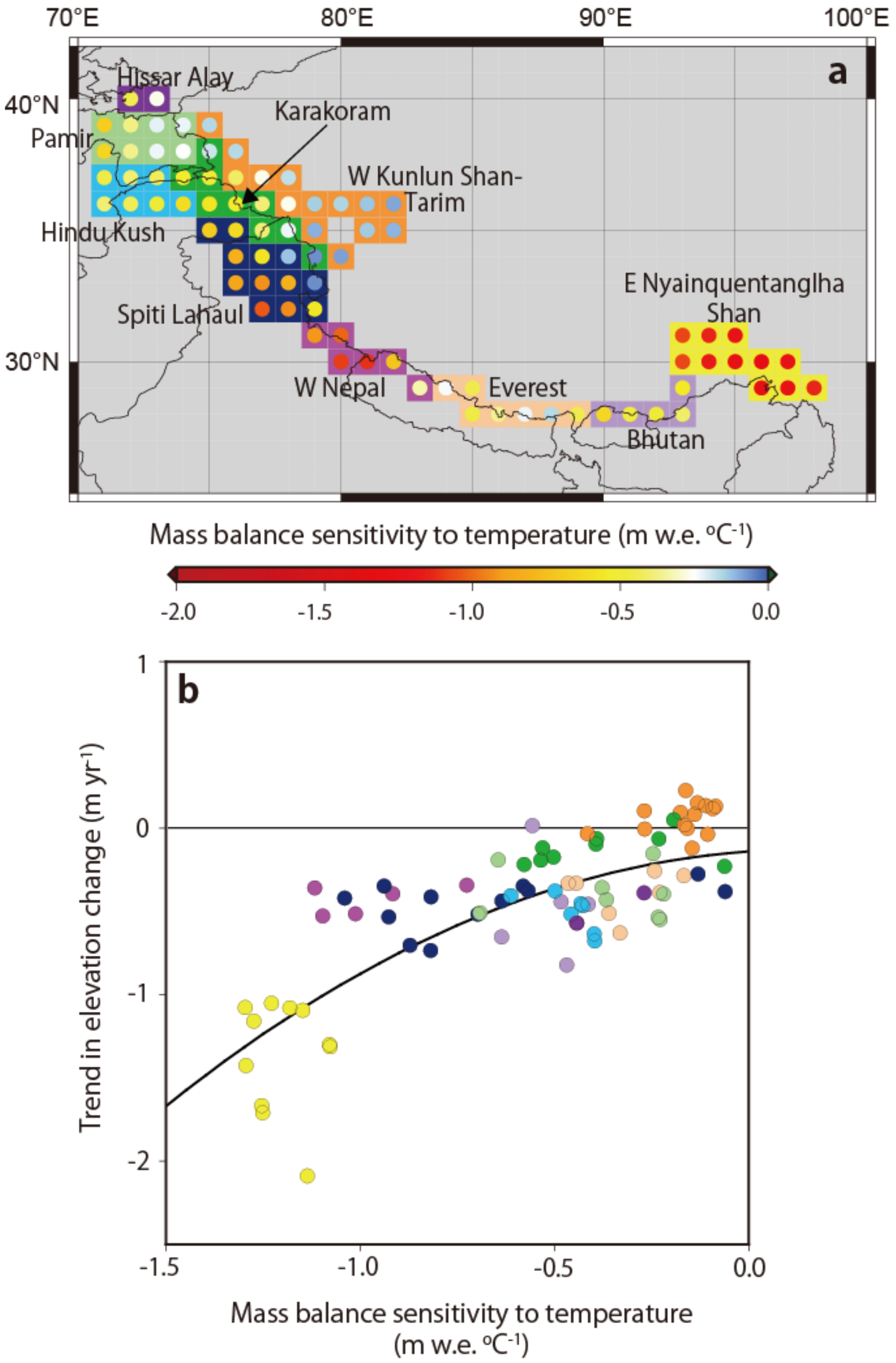

Fig. S5: MBS for 10 sub-regions. **a**: Distribution of MBS for 10 sub-regions, which are divided from the domains in Kääb et al.[2]. **b**: Relationship between the MBS and TEC[2] coloured by sub-regions depicted in **a**. The regression line is the same as that shown in Fig. 2. These figures were created using 'The Generic Mapping Tools' (http://gmt.soest.hawaii.edu/), Version 5.1.0. and were edited using Adobe Illustrator CS6 Version 16.0.0.

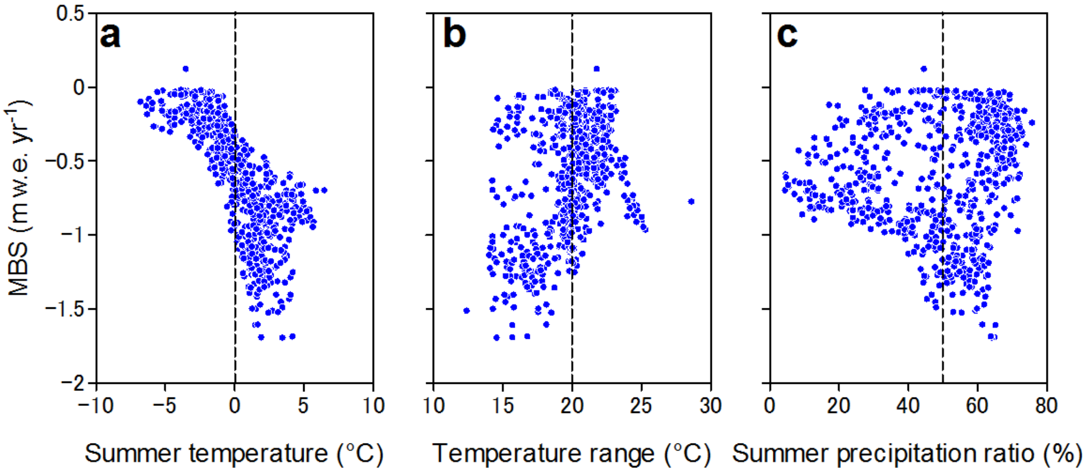

Fig. S6: Relationship between MBS and explanatory factors, **a.** summer temperature, **b.** annual range in monthly temperature (temperature range), and **c.** summer precipitation ratio. Dashed vertical lines indicate the threshold values used in Fig. 3.

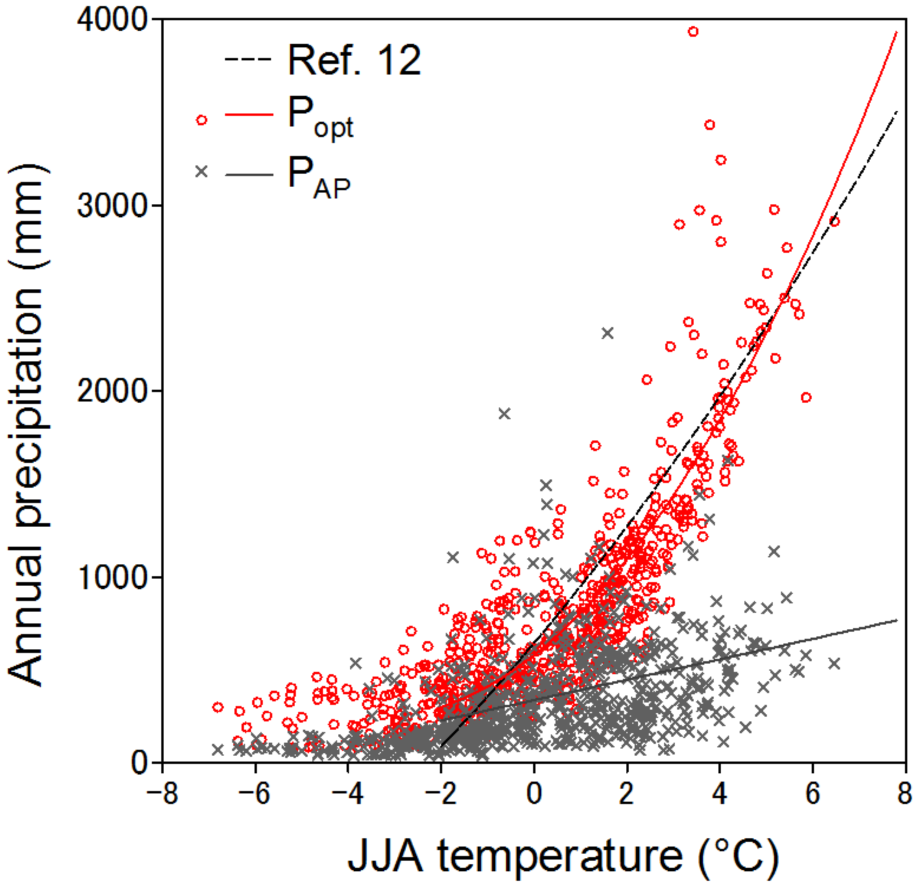

Fig. S7: Relationship between summer June-July-August (JJA) mean air temperature and annual precipitation at average median elevations. Annual precipitation from original APHRODITE data are averaged for the period 1979-2007 (grey crosses) and optimized in this study. Fitting curves are plotted with colours corresponding to the original and optimized data. The black dashed curve is from ref. 12.

Tables:

Table S1 Statistical summary of fitting relationships between MBS and TECs. TECs are based on Kääb *et al.* (2), Gardner *et al.* (1) for all of high-mountain Asia (HMA), and Gardner *et al.* (1) with high overlapping ratios. *n*, *r,* and *p* are sample number, correlation coefficient, and significant level, respectively.

Data source for TECs	n	*r*	*p*	Equation of fitting curve and determination coefficient (R^2) (x = MBS, y =TECs)
Kääb et al.[2]	81	0.76	< 0.001	$y = -0.595x^2 + 0.146x - 0.110$ ($R^2 = 0.61$)
Gardner et al.[1] for all of HMA	277	0.41	< 0.001	$y = 0.805x^2 + 1.727x - 0.294$ ($R^2 = 0.19$)
Gardner et al.[1] with high overlapping ratios (> 0.8)	25	0.61	< 0.005	$y = 1.414x^2 + 2.382x + 0.396$ ($R^2 = 0.43$)

Table S2 Summary of multi-regression analysis on MBS for each 0.5º grid in the two cases, four and three explanatory variables. First, we assumed five explanatory variables; summer temperature ($\boldsymbol{S_t}$), annual precipitation, annual range of monthly air temperature ($\boldsymbol{T_r}$), summer precipitation ratio ($\boldsymbol{S_{pr}}$), and summer solar radiation ($\boldsymbol{S_s}$) for MBS. However, the annual precipitation is highly correlated with summer temperature (r = 0.77) so we exclude the annual precipitation to avoid multicollinearity. The t value is the ratio of the sample regression coefficient to its standard error, and a larger absolute value indicates a stronger correlation with MBS. The p value indicates significant level, where values of 0.05 indicate a 95% probability that the variable has some effect.

	Determination coefficient (R^2) with ***MBS***	Four explanatory variables		Three explanatory variables	
		t	p	t	p
$\boldsymbol{S_t}$ (ºC)	0.58	–27.90	< 0.001	–33.74	< 0.001
$\boldsymbol{T_r}$ (ºC)	0.15	5.19	< 0.001	12.49	< 0.001
$\boldsymbol{S_{pr}}$ (%)	0.01	–4.63	< 0.001	–6.23	< 0.001
$\boldsymbol{S_s}$ (W m^{-2})	0.30	3.49	< 0.05	-	-
Determination coefficient (R^2)		0.698		0.692	
Multiple regression equation for ***MBS*** (m w.e. yr^{-1})		$\boldsymbol{MBS}$= –0.121$\boldsymbol{S_t}$ + 0.031$\boldsymbol{T_r}$ – 0.00262$\boldsymbol{S_{pr}}$ + 0.00125$\boldsymbol{S_s}$ –1.40		$\boldsymbol{MBS}$= –0.129$\boldsymbol{S_t}$ + 0.0477$\boldsymbol{T_r}$–0.00332$\boldsymbol{S_{pr}}$ –1.37	
